# Health Tract, No. 28

**Published:** 1862-01

**Authors:** 


### HEALTH TRACT, No. 28.

### AVERTING DISEASE.

(*From Hall's Journal of Health, New-York.* $1 *a Year.*)

PAIN is a blessing; it is the great life-preserver; it is the sleepless, faithful sentinel which gives prompt warning that harm is being done. Pain is the result of pressure on or against a nerve; that pressure is made by a blood-vessel, for there is no nerve without a blood-vessel in close proximity. In health, each blood-vessel is moderately full; but the very moment disease, or harm, or violence, by blow or cut or otherwise, comes to any part of the body, nature becomes alarmed as it were, and sends more blood there to repair the injury—much more than is usually required; that additional quantity distends the blood-vessels, presses against a nerve, and gives disquiet or actual pain. In these cases this increased quantity of blood is called "inflammation." Again, if a man eats too much, or is constipated, or by some other means makes his blood impure, it becomes thickened thereby, and does not flow through its channels as freely as it should; hence it accumulates, dams up, congests, distending the veins, which in their turn make pressure on some adjoining nerve, and give dull pain, as headache. This congestion in the arteries gives a sharp, pricking pain.

Pain, then, is the result of more blood being determined to the part where that pain is, than naturally belongs to it. The evident alternative is to diminish the quantity of blood, either at the point of ailment or in the body in general. Thus it is that a mustard-plaster applied near a painful spot, by withdrawing the blood to itself, gives instantaneous relief. Opening a vein will do the same thing; and so, but not as expeditiously, will any purgative medicine, because that by all these things, by diminishing the amount of fluid as to the whole body, each particular part is proportionably relieved. On the same principle is, it that a "good sweat" is "good" for any pain, and affords more or less relief. Friction does the same, even if it is performed with so soft a thing as the human hand, for any rubbing reddens, that is, attracts blood to the part rubbed, and thus diminishes the pain at the spot where there is too much blood.

1. The instant we become conscious of any unpleasant sensation in the body, eat nothing. 2. Keep warm. 3. Be still.

These are applicable and safe in all cases; sometimes a more speedy result is attained if, instead of being quiet, the patient would, by moderate, steady exercise, keep up a gentle perspiration for several hours. In many cases, this remedy will become more and more efficient, with increasing intervals for need of its application, until at length a man is not sick at all, and life goes out like the snuff of a candle or as gently as the dying embers on the hearth.

